# Functionally Selective Signaling for Morphine and Fentanyl Antinociception and Tolerance Mediated by the Rat Periaqueductal Gray

**DOI:** 10.1371/journal.pone.0114269

**Published:** 2014-12-11

**Authors:** Michael M. Morgan, Rachel A. Reid, Kimber A. Saville

**Affiliations:** Department of Psychology, Washington State University Vancouver, Vancouver, Washington, 98686, United States of America; University of Medicine & Dentistry of NJ - New Jersey Medical School, United States of America

## Abstract

Functionally selective signaling appears to contribute to the variability in mechanisms that underlie tolerance to the antinociceptive effects of opioids. The present study tested this hypothesis by examining the contribution of G protein-coupled receptor kinase (GRK)/Protein kinase C (PKC) and C-Jun N-terminal kinase (JNK) activation on both the expression and development of tolerance to morphine and fentanyl microinjected into the ventrolateral periaqueductal gray of the rat. Microinjection of morphine or fentanyl into the periaqueductal gray produced a dose-dependent increase in hot plate latency. Microinjection of the non-specific GRK/PKC inhibitor Ro 32-0432 into the periaqueductal gray to block mu-opioid receptor phosphorylation enhanced the antinociceptive effect of morphine but had no effect on fentanyl antinociception. Microinjection of the JNK inhibitor SP600125 had no effect on morphine or fentanyl antinociception, but blocked the expression of tolerance to repeated morphine microinjections. In contrast, a microinjection of Ro 32-0432 blocked the expression of fentanyl, but not morphine tolerance. Repeated microinjections of Ro 32-0432 blocked the development of morphine tolerance and inhibited fentanyl antinociception whether rats were tolerant or not. Repeated microinjections of SP600125 into the periaqueductal gray blocked the development of tolerance to both morphine and fentanyl microinjections. These data demonstrate that the signaling molecules that contribute to tolerance vary depending on the opioid and methodology used to assess tolerance (expression vs. development of tolerance). This signaling difference is especially clear for the expression of tolerance in which JNK contributes to morphine tolerance and GRK/PKC contributes to fentanyl tolerance.

## Introduction

Opioids such as morphine and fentanyl are the most commonly used and effective drugs to treat severe pain. Unfortunately, tolerance to the analgesic effects of opioids can occur following a single injection and can result in a 10-fold escalation in the dose needed to relieve pain [1], [2]. Tolerance to morphine is easy to induce in laboratory animals, and thousands of studies examining the neural mechanisms underlying tolerance have been undertaken. Despite this effort, there is no coherent understanding of the molecular changes that cause opioid tolerance.

**Figure 1:**
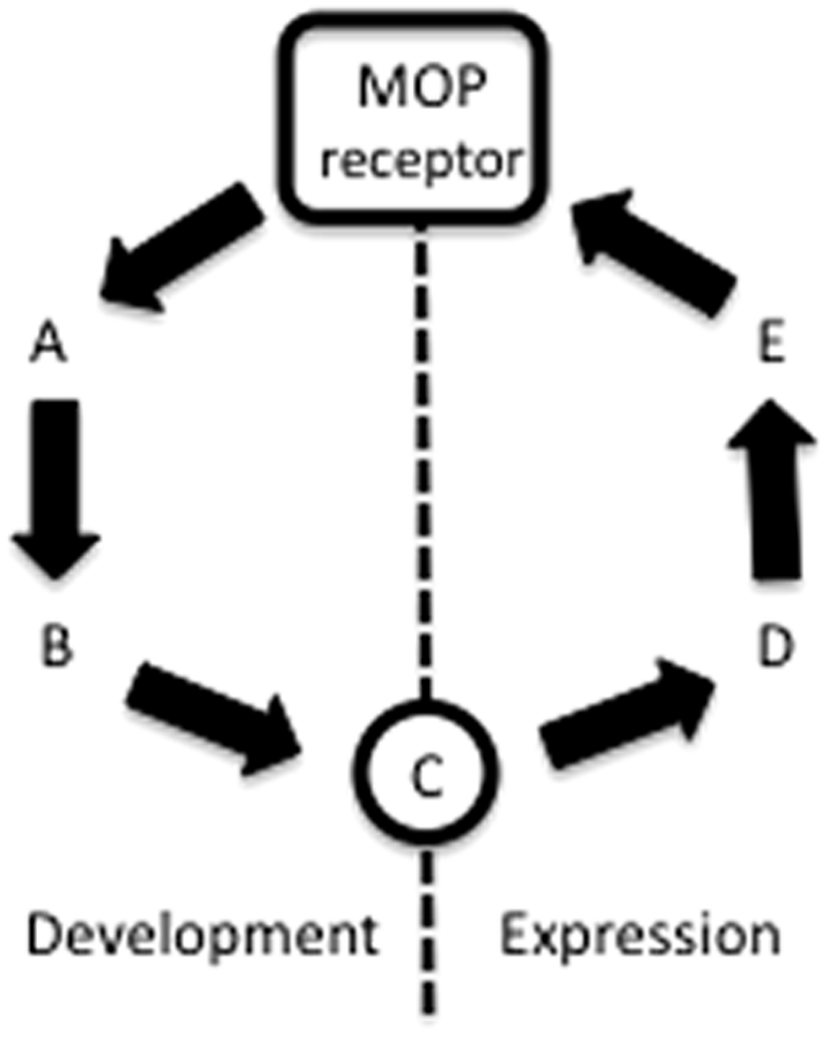
Model of MOPr signaling showing that distinct molecules contribute to the development and expression of opioid tolerance. Tolerance could be caused by a change anywhere along the signaling pathway. If this change occurs at point C in the model, then repeated co-administration of an opioid with a drug that blocks signaling at points A, B, or C will prevent the development of tolerance. Once tolerance has developed, blocking signaling at points A or B will have no effect on the expression of tolerance because signaling at point C is already altered. However, a drug that blocks the enhanced signaling from points C, D, or E will block the expression of tolerance.

The primary problem is that there are multiple mechanisms for opioid tolerance and the contribution of a specific mechanism varies with subtle differences in experimental design. For example, different mechanisms are engaged in different parts of the nervous system as demonstrated by the involvement of NMDA receptors in tolerance when morphine is administered to the spinal cord, but not to the periaqueductal gray (PAG) [3], [4]. Second, the signaling molecules involved in tolerance may differ depending on whether the development or expression of tolerance is assessed (Fig. 1). Third, different molecules contribute to tolerance to different opioids. Tolerance occurs to morphine, but not fentanyl in G protein-coupled receptor kinase (GRK) knock out mice, whereas blocking C-Jun N-terminal kinase (JNK) disrupts tolerance to a single injection of morphine, but not fentanyl [5]. Others have shown that pharmacological disruption of GRK signaling prevents the expression of tolerance to DAMGO, but not morphine or fentanyl [6].

The present study addresses these methodological issues by examining both the development and expression of tolerance to microinjections of morphine or fentanyl into the ventrolateral PAG. The ventrolateral PAG is known to contribute to both antinociception [7], [8] and tolerance [9]–[11] to morphine and fentanyl, and microinjections into the PAG limits drug action while also allowing neural changes to be linked to behavior. The contribution of GRK/PKC and JNK signaling to this antinociception is particularly interesting because activation of GRK causes mu-opioid receptor (MOPr) phosphorylation which terminates the antinociceptive signaling driven by G-proteins [12]–[14] and JNK signaling inhibits MOPr gene expression [15]. Enhancement of MOPr phosphorylation or activation of JNK could contribute to tolerance by reducing MOPr signaling from the plasma membrane. The present study tested this hypothesis by assessing the effect of blocking GRK and JNK signaling on nociception, antinociception, and the expression and development of tolerance to repeated microinjections of morphine or fentanyl into the ventrolateral PAG. The present data support the hypothesis that distinct molecular signaling pathways underlie antinociception and tolerance to morphine and fentanyl.

## Materials and Methods

### Subjects

Data were collected from 257 adult male Sprague-Dawley rats purchased from Harlan Laboratories (Livermore, CA). Rats were anesthetized with pentobarbital (60 mg/kg, i.p) and implanted with a guide cannula (23 gauge; 9 mm long) aimed at the ventrolateral PAG using a stereotaxic apparatus (AP: +1.7 mm, ML: 0.6 mm, DV: −4.6 mm from lambda). Dental cement was used to secure the guide cannula to two screws placed in the skull. Following surgery, a stylet (9 mm) was inserted into the guide cannula and the rat was allowed to recover under a heat lamp until awake.

Rats were housed individually or in pairs in a room maintained on a 12∶12 hr reverse light cycle (lights off at 7:00 AM). Food and water were available at all times, except during testing. Each rat was handled daily for at least one week between surgery and the initiation of testing. Rats weighed between 192 and 353 g at the start of the experiment (median  = 268 g).

### Ethical Statement

All procedures were approved by the Washington State University Animal Care and Use Committee (Permit Number 2156) and conducted in accordance with the International Association for the Study of Pain's Policies on the Use of Animals in Research. The number of rats used was kept to a minimum by using cumulative doses to generate dose-response curves. Potential suffering was minimized by assessing nociception with the hot plate test (see below).

### Microinjections and Behavioral Testing

Morphine sulfate, fentanyl citrate, the GRK/Protein Kinase C (PKC) inhibitor Ro 32-0432 [16], the JNK inhibitor SP600125 [17] or the appropriate vehicle were administered into the ventrolateral PAG through a 31-gauge injection cannula that extended 2 mm beyond the guide cannula. All drugs were purchased from Sigma-Aldrich (St. Louis, MO) except morphine, which was a gift from the National Institute on Drug Abuse. Saline was the vehicle for morphine and fentanyl, whereas the vehicle for Ro 32-0432 was a 7∶2∶1 ratio of saline/Cremophor/DMSO, and the vehicle for SP600125 was an 8∶1∶1 ratio of saline/Tween/DMSO. Microinjections were administered in a volume of 0.4 µL at a rate of 0.1 µL/10 s. The injection cannula remained in place for 20 s after the injection to minimize backflow of the drug up the cannula tract. Immediately following the microinjection, the stylet was replaced and the rat was returned to its home cage. A sham injection in which the injector was inserted into the guide cannula without drug administration was conducted 24 hours prior to the first microinjection. The purpose of this sham injection was to habituate the rat to the injection procedure and prevent confounds caused by mechanical activation of neurons on the test day.

The doses of morphine (5 µg/0.4 µL) and fentanyl (3 µg/0.4 µL) selected for the induction of tolerance were slightly higher than the half maximal dose (D_50_) for antinociception following microinjection into the ventrolateral PAG [7], [9]. The doses and pretreatment time (20 min) for Ro 32-0432 (400 ng/0.4 µl) and SP600125 (100 ng/0.4 µL) were selected based on conversion from systemic or intracerebroventricular doses [5], [6] and preliminary testing.

The hot plate test was used to assess nociception because it is sensitive to opioid microinjections into the PAG and can be applied repeatedly without damaging the skin [9], [10]. Rats were placed on a 52.5°C hot plate and the latency to lick a hind paw was measured. The rat was removed from the plate if no response occurred within 50 s.

### Procedure

Rats were injected with morphine, fentanyl, or saline into the ventrolateral PAG twice daily for two consecutive days (Trials 1–4). Injections were administered at approximately 10:00 and 16:00 each day. Given the difference in time to maximal antinociception following microinjection of morphine and fentanyl [7], nociception was assessed 30 min following the first microinjection of morphine and 3 min following the first microinjection of fentanyl. No hot plate testing was conducted following microinjections on Trials 2–4 to prevent the development of behavior tolerance as a result of repeated testing [18], [19].

The potency of morphine or fentanyl was assessed 18 hours after the microinjection on Trial 4 using a cumulative dosing procedure. Baseline nociception was assessed using the hot plate test followed by microinjections of cumulative doses of morphine (1, 2.2, 4.6, 10, & 22 µg/0.4 µl) or fentanyl (0.46, 1.0, 2.2, 4.6, & 10 µg/0.4 µl) into the ventrolateral PAG [10]. Morphine was administered every 20 min and hot plate latency was measured 15 min after each injection until the hot plate latency reached 50 s or the highest dose had been administered (a cumulative dose of 22 µg). Given the rapid onset and short duration of action of fentanyl following microinjection into the ventrolateral PAG [7], fentanyl was injected every 4 min and hot plate latency was measured 2 min after each injection until the hot plate latency reached 50 s or the highest dose had been administered (a cumulative dose of 10 µg).

#### Effect of GRK/PKC and JNK Inhibition on Nociception

Rats treated with saline on Trials 1–4 were used to determine the effects of Ro 32-0432 and SP600125 on nociception. Eighteen hours after the last saline injection on Trial 4, hot plate latency was assessed before and 15 min after microinjection of Ro 32-0432 (400 ng/0.4 µl), SP600125 (100 ng/0.4 µl), or the appropriate vehicle into the ventrolateral PAG.

#### Effect of GRK/PKC and JNK Inhibition on Antinociception

Following the baseline tests described above, the effects of Ro 32-0432 and SP600125 on morphine and fentanyl antinociception were assessed. Rats were injected with saline into the ventrolateral PAG on Trials 1–4 so they received the same number of injections as rats made tolerant to morphine or fentanyl, but were naïve to Ro 32-0432, SP600125, morphine, and fentanyl. Cumulative doses of morphine or fentanyl were administered on Trial 5 starting 20 min after microinjection of Ro 32-0432 or SP600125 into the ventrolateral PAG. The effect of each protein inhibitor (Ro 32-0432 or SP600125) on morphine and fentanyl potency was compared to rats injected with vehicle.

#### Effect of GRK/PKC and JNK Inhibition on the Expression of Tolerance

The contribution of GRK/PKC and JNK to the expression of tolerance was assessed in rats treated with morphine or fentanyl on Trials 1–4. Tolerance was defined as a rightward shift in the dose-response curve on Trial 5 compared to rats treated with saline on Trials 1–4. On Trial 5, Ro 32-0432, SP600125, or the appropriate vehicle was injected 20 min prior to administration of cumulative doses of morphine or fentanyl into the ventrolateral PAG. The blockers were only injected prior to the final test in order to assess the effect of blocking these proteins during the expression as opposed to the development of tolerance. A leftward shift in the opioid dose response curve in rats treated with an inhibitor compared to rats treated with vehicle indicates that GRK/PKC or JNK contributes to opioid tolerance.

#### Effect of GRK/PKC and JNK Inhibition on the Development of Tolerance

Ro 32-0432, SP600125, or vehicle was microinjected into the ventrolateral PAG 20 minutes prior to each opioid or saline microinjection on Trials 1–4 to determine whether blocking GRK/PKC or JNK activation prevents the development as opposed to the expression of opioid tolerance. Tolerance was assessed on Trial 5 in the absence of the blockers by microinjecting cumulative doses of morphine or fentanyl into the ventrolateral PAG.

### Histology

**Figure 2:**
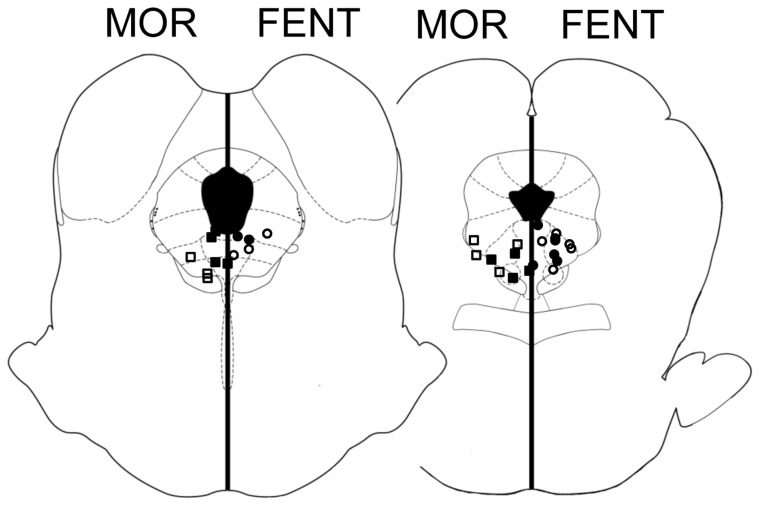
Location of representative microinjection sites in the ventrolateral PAG. Morphine injections (filled squares) are shown on the left and fentanyl injections (filled circles) on the right even though all injections were administered on the right side of the PAG. The effect of the opioid was compared between rats receiving repeated opioid (closed symbols) and saline injections (open symbols). All injections were located between coronal sections 0.98 and 1.56 from the interaural line [20].

Immediately following testing, rats were exposed to a lethal dose of Halothane. The brain was removed and placed in formalin (10%) for at least 48 hours. Coronal sections through the caudal PAG (100 µm) were made with a vibratome to determine the location of the injection cannula. Only injection sites located within or immediately adjacent to the ventrolateral PAG [20] were included in data analysis (Fig. 2).

### Data Analysis

Hot plate latency data from Trial 1 were analyzed using ANOVA or t-test as appropriate. Tolerance was assessed by comparing shifts in the opioid dose-response curves in rats pretreated with morphine, fentanyl, or saline as assessed by the half-maximal antinociceptive effect (D_50_) [21]. The D_50_ value for each group was calculated using non-linear regression (GraphPad Prism) with the lower limit set at the mean baseline hot plate latency and the upper limit set at the cutoff hot plate latency (50 s). Changes in D_50_ values were assessed using ANOVA (GraphPad Prism).

## Results

### Nociception

There was no effect on baseline nociception following inhibition of GRK/PKC (F(1,24)  = 0.039, n.s.) or JNK (F(1,28)  = 0.889, n.s.) activation in the PAG. Mean hot plate latency went from 14.3±1.1 to 14.9±1.8 s following Ro 32-0432 microinjection into the ventrolateral PAG and from 14.4±1.0 to 15.5±1.2 s following vehicle microinjection. Mean hot plate latency went from 13.15±1.1 to 12.4±1.6 s following microinjection of SP600125 into the ventrolateral PAG and from 12.8±1.0 to 13.8±1.3 s following microinjection of the SP600125 vehicle. These data indicate that subsequent changes in morphine or fentanyl antinociception caused by blocking GRK/PKC or JNK are not caused by a shift in baseline nociception.

### Antinociception

**Figure 3:**
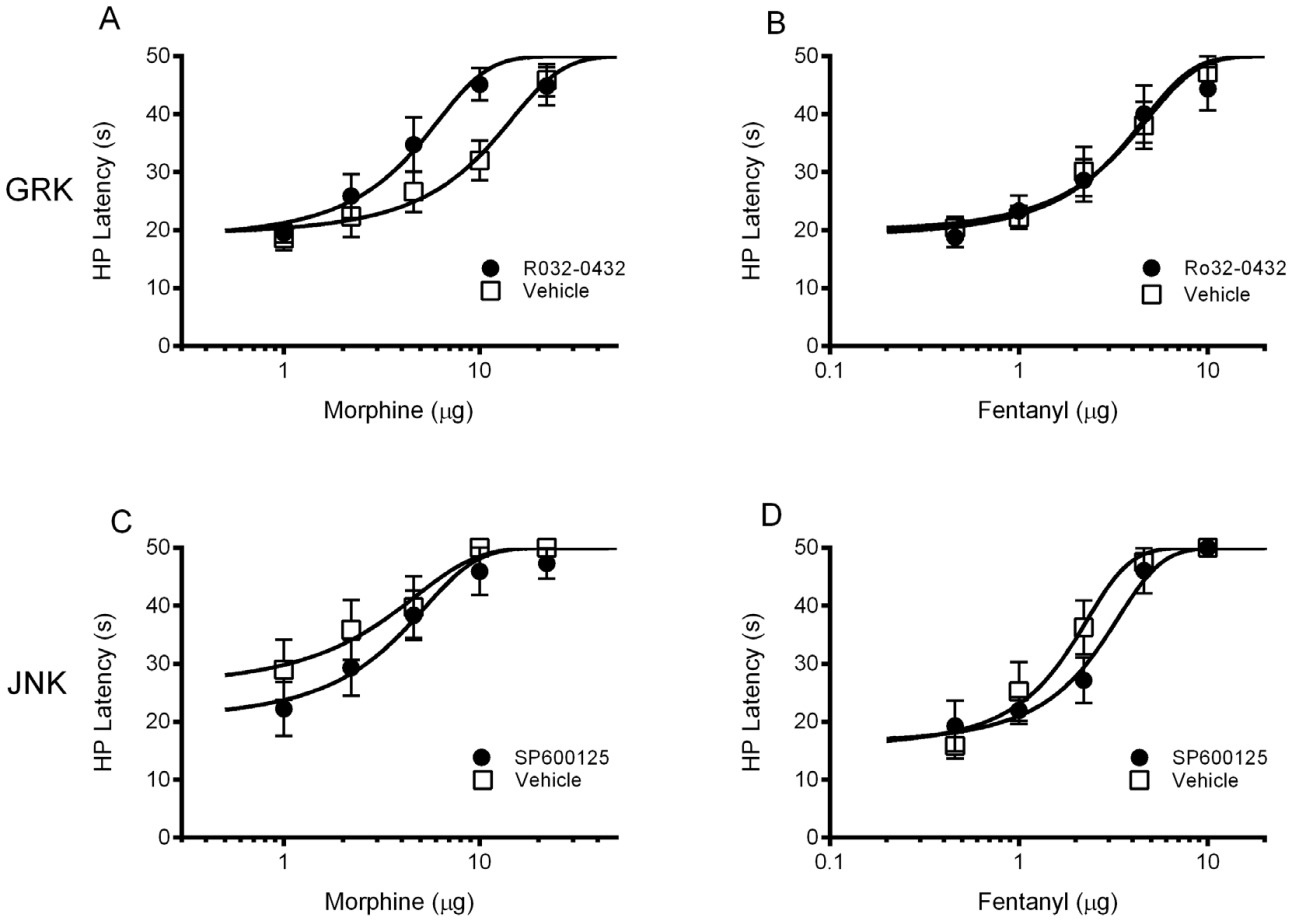
Analysis of GRK/PKC and JNK inhibition on morphine and fentanyl antinociception. Rats were injected with the GRK/PKC inhibitor Ro 32-0432 (400 ng/0.4 µl), the JNK inhibitor SP600125 (100 ng/0.4 µL), or the appropriate vehicle into the ventrolateral PAG 20 min before microinjection of cumulative doses of morphine or fentanyl (N = 7–11/condition). Morphine or fentanyl administration produced a dose dependent increase in hot plate (HP) latency. Microinjection of Ro 32-0432 into the ventrolateral PAG enhanced the antinociceptive effect of morphine (A) as indicated by a leftward shift in the morphine dose-response curve, but had no effect on fentanyl antinociception (B). Neither morphine (C) nor fentanyl (D) antinociception were altered by microinjection of SP600125 into the ventrolateral PAG.

Microinjection of Ro 32-0432 into the ventrolateral PAG to block GRK/PKC activation enhanced the antinociceptive effect of morphine, but had no effect on fentanyl antinociception (Figs. 3A & B). A significant leftward shift in the morphine dose response curve was evident in rats treated with Ro 32-0432 compared to vehicle treated controls (F(1,101)  = 11.66, p = .0009). The fentanyl dose-response curves were nearly identical whether rats were treated with Ro 32-0432 or vehicle (F(1,96)  = 0.001, p = .97). The enhancement of morphine antinociception by Ro 32-0432 administration is consistent with prolonged G-protein signaling as a result of blocking MOPr phosphorylation [14].

There was no significant inhibition of morphine (F(1,76)  = 2.641, p = .1085) or fentanyl (F(1,71)  = 3.355, p = .0712) antinociception following microinjection of the JNK inhibitor SP600125 into the ventrolateral PAG. The morphine and fentanyl dose-response curves are similar whether rats were treated with SP600125 or vehicle (Figs. 3C & D). The lack of effect of JNK inhibition on morphine or fentanyl antinociception is consistent with previous research in mice [5].

### Expression of Tolerance

**Figure 4:**
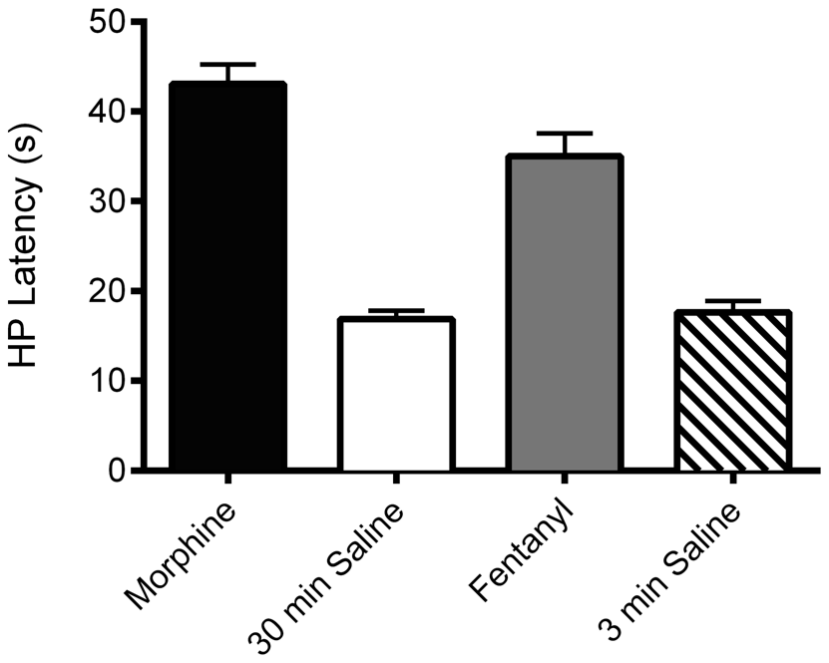
Microinjection of morphine and fentanyl into the ventrolateral PAG produced antinociception on Trial 1. Despite different doses and test times, microinjection of morphine (tested 30 min after a dose of 5 µg, N = 32) or fentanyl (tested 3 min after a dose of 3 µg, N = 33) produced a significant increase in hot plate (HP) latency compared to saline treated controls tested 30 or 3 min after the microinjection (F(3,136) = 51.60; p = .0001).

Microinjection of morphine or fentanyl into the ventrolateral PAG on Trial 1 caused a significant increase in hot plate latency compared to saline-pretreated animals as expected (Fig. 4; F(3,136)  = 51.60; p = .0001). The magnitude of antinociception produced by these doses and test times resulted in a slightly greater antinociception for morphine (5 µg/0.4 µL at 30 min) compared to fentanyl (3 µg/0.4 µL at 3 min) treated rats (*t*(63)  = 2.376, p = .02), but this difference was small compared to the magnitude of antinociception in both groups (Fig. 4). The same doses were injected on Trials 2–4, but nociception was not assessed following these injections to prevent the development of behavioral tolerance from repeated testing [18], [19]. Each of these groups was divided into two conditions on Trial 5 to determine the effects of Ro 32-0432 and SP600125 on the expression of morphine and fentanyl tolerance.

Tolerance was assessed on Trial 5, 18 hours after the Trial 4 injection. There was no significant difference in baseline hot plate latency immediately prior to Trial 5 injections whether rats had received morphine (13.0±0.6 s), fentanyl (14.5±0.8 s), or saline (14.3±0.5 s) microinjections on Trials 1–4 (F(2,134)  = 1.434, n.s.). These data show that there was no lasting effect of repeated morphine or fentanyl microinjections on nociception when assessed one day later.

Microinjection of cumulative doses of morphine or fentanyl into the ventrolateral PAG produced a dose-dependent increase in hot plate latency in all groups. The potency of morphine antinociception was reduced in rats injected with morphine on Trials 1–4 compared to saline treated rats as would be expected with the development of tolerance [F(2,119)  = 3.935, p = .0221 and F(2,109)  = 7.755, p = .0007, for the Ro 32-0432 and SP600125 experiments, respectively; Table 1]. Likewise, the potency of fentanyl antinociception was reduced in rats treated with fentanyl on Trials 1–4 compared to saline treated rats [F(2,124)  = 4.933, p = .0087 and F(2,114)  = 3.950, p = .022 for the Ro 32-0432 and SP600125 experiments, respectively; Table 1].

**Table 1:** Effect of GRK/PKC and JNK inhibition on the Expression of Tolerance.

Protein	Trials 1–4	Trail 5	Morphine D_50_ (N)	Fentanyl D_50_ (N)
	Saline	Vehicle	4.5 µg (17)	2.3 µg (17)
GRK/PKC	Opioid	Vehicle	9.9 µg (8)	3.9 µg (7)
	Opioid	Ro 32-0432	*15.5 µg (7)	*2.0 µg (9)
JNK	Opioid	Vehicle	8.0 µg (7)	3.1 µg (10)
	Opioid	SP600125	*3.9 µg (9)	2.5 µg (7)

Notes: N =  sample size.

*Denotes a significant difference from the vehicle control.

**Figure 5:**
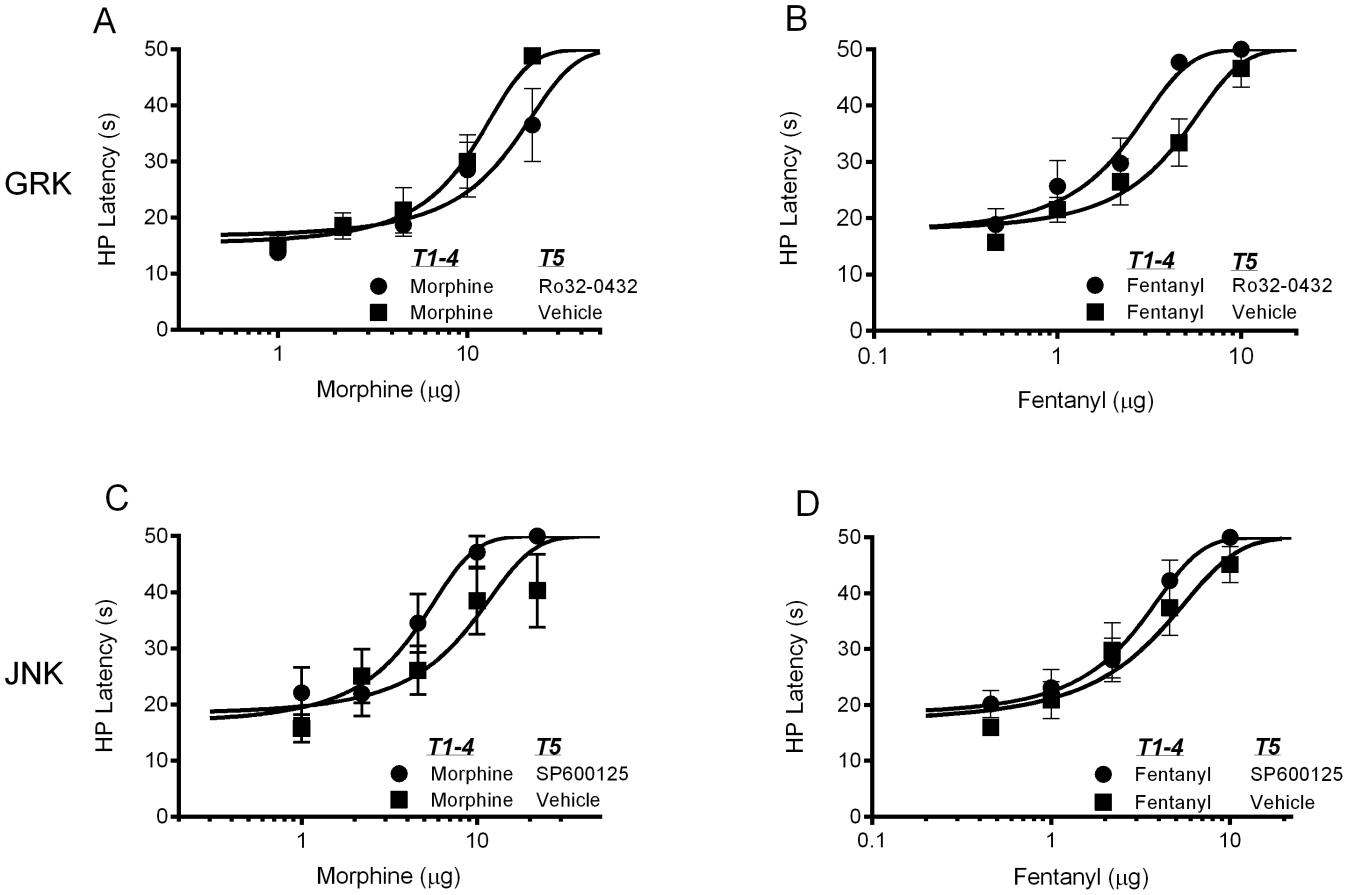
GRK/PKC contributes to the expression of fentanyl tolerance, and JNK contributes to the expression of morphine tolerance. A) Microinjection of the GRK/PKC inhibitor Ro 32-0432 (400 ng/0.4 µl) into the ventrolateral PAG 20 min prior to administration of cumulative doses of morphine on Trial 5 enhanced the expression of morphine tolerance (F(1,71)  = 5.061, p = .0276). B) In contrast, microinjection of Ro 32-0432 on Trial 5 reversed the expression of fentanyl tolerance (F(1,76)  = 10.55, p = .0017). C) Microinjection of the JNK inhibitor SP600125 (100 ng/0.4 µL) on Trial 5 reversed the expression of morphine tolerance (F(1,76)  = 4.436, p = .0385), but D) had no effect on the expression of fentanyl tolerance (F(1,81)  = 0.880, p = .351).

Microinjection of the GRK/PKC inhibitor Ro 32-0432 into the ventrolateral PAG enhanced the expression of morphine tolerance (Fig. 5A), but reversed fentanyl tolerance (Fig. 5B). In contrast, microinjection of the JNK inhibitor SP600125 attenuated the expression of morphine tolerance as indicated by a leftward shift in the morphine dose response curve (Fig. 5C), but had no effect on the fentanyl dose response curve (Fig. 5D). The D_50_ value for each condition is presented in Table 1. These data demonstrate ligand-biased activation of distinct signaling molecules in the expression of tolerance: GRK/PKC activation contributes to the expression of fentanyl tolerance and JNK activation contributes to the expression of morphine tolerance.

### Development of Tolerance

**Figure 6:**
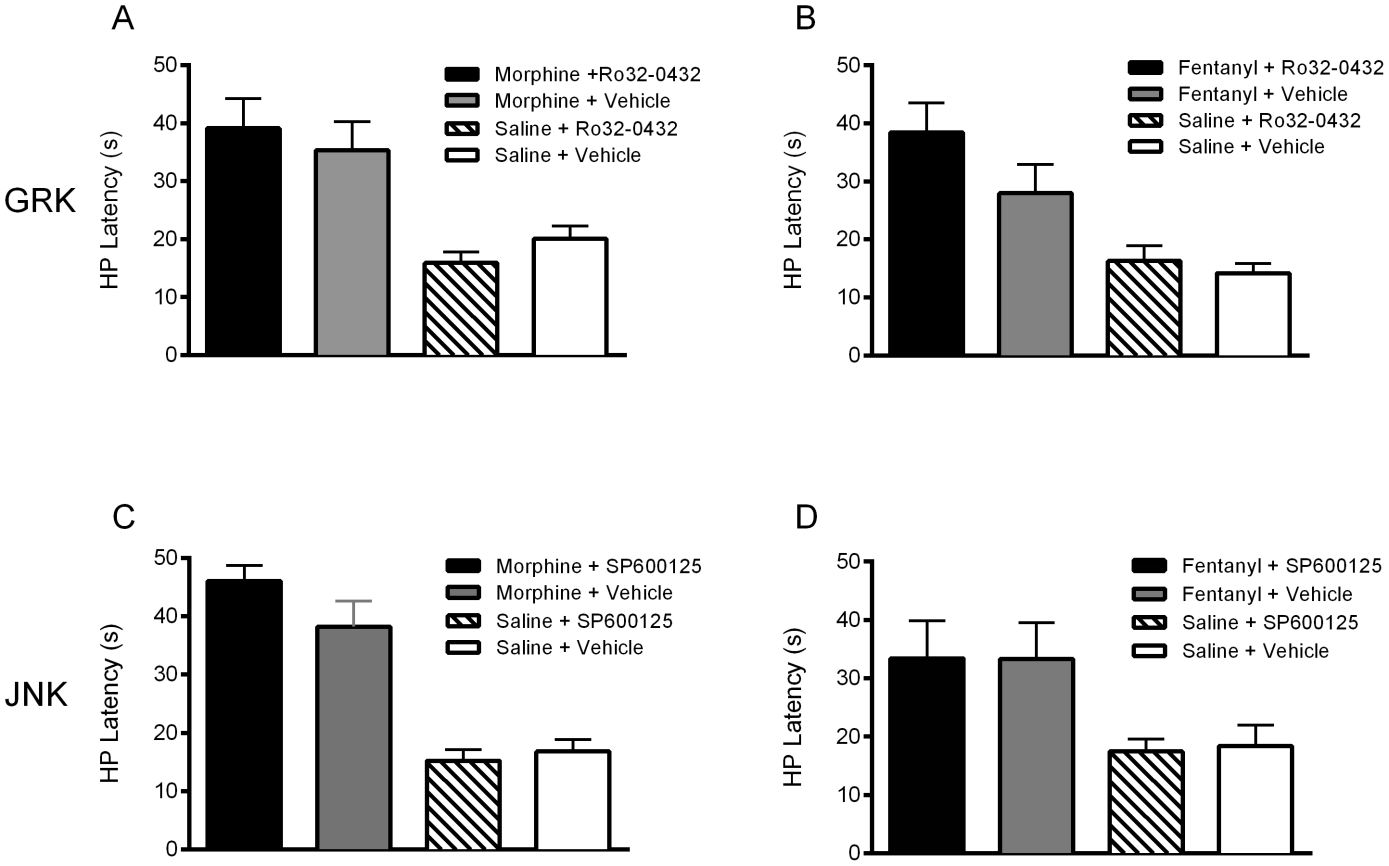
The antinociceptive effects of microinjecting morphine or fentanyl into the ventrolateral PAG on Trial 1 were not altered by blocking activation of GRK/PKC or JNK. A) Microinjection of morphine produced an increase in hot plate latency compared to vehicle treated controls (F(3,32)  = 8.592, p = .0003) whether rats were pretreated with the GRK/PKC inhibitor Ro 32-0432 (400 ng/0.4 µl) or not (Bonferroni, t = 0.689, n.s.). B) Likewise, fentanyl antinociception (F(3,29)  = 7.661, p = .0008) was not altered by pretreatment with Ro 32-0432 (t = 1.882, n.s.). C) Microinjection of morphine produced an increase in hot plate latency compared to vehicle treated controls (F(3,29)  = 29.67, p = .0001) whether rats were pretreated with SP600125 (100 ng/0.4 µL) or not (t = 1.942, n.s.). D) Likewise, fentanyl antinociception (F(3,28)  = 3.194, p = .041) was not altered by SP600125 pretreatment (t = 0.008, n.s.).

Rats received repeated injections of Ro 32-0432, SP600125, or vehicle along with morphine, fentanyl, or saline into the ventrolateral PAG on Trials 1–4 to determine whether GRK/PKC and JNK contribute to the development of tolerance. The antinociception produced by microinjecting morphine or fentanyl into the ventrolateral PAG on Trial 1 was evident regardless of pretreatment with Ro 32-0432, SP600125, or vehicle (Fig. 6). In no case did repeated microinjection of Ro 32-0432 or SP600125 alter the antinociception evoked by morphine or fentanyl compared to vehicle treated controls given morphine or fentanyl. Each rat received the same drug combination for Trials 1–4, but no behavioral testing was conducted on Trials 2–4.

**Figure 7:**
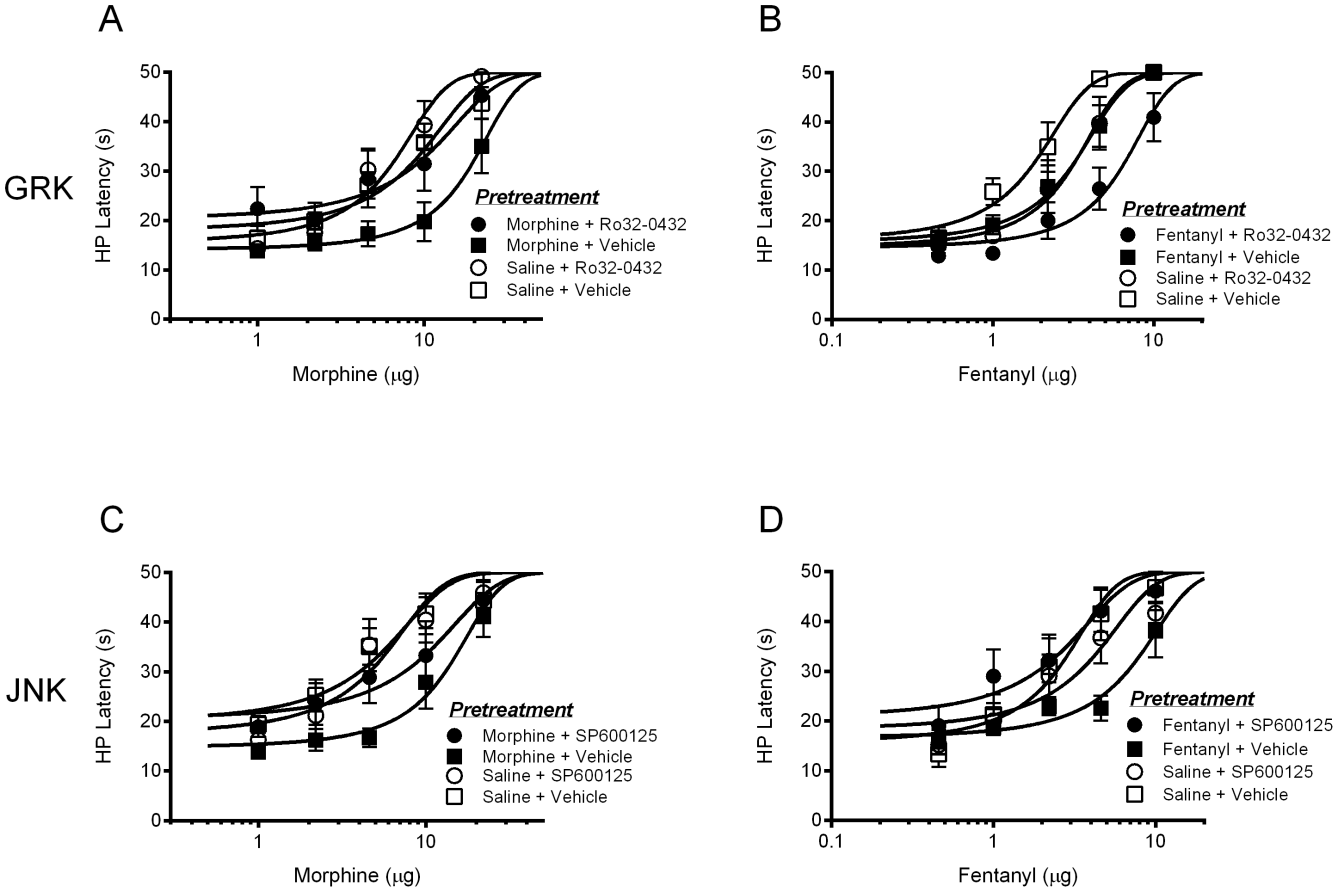
Contribution of GRK/PKC and JNK to the development of morphine and fentanyl tolerance. A) Repeated microinjections of morphine into the ventrolateral PAG caused tolerance as evident by a rightward shift in the morphine dose response curve (F(3,157)  = 3.689, p = .0043). Administration of the GRK/PKC inhibitor Ro 32-0432 (400 ng/0.4 µl) with morphine on Trials 1–4 prevented the development of morphine tolerance (p<.05). B) Repeated microinjections of fentanyl into the ventrolateral PAG also caused tolerance (F(3,142)  = 17.10, p = .0001). Administration of Ro 32-0432 on Trials 1–4 caused a rightward shift in the fentanyl dose-response curve whether rats were made tolerant to fentanyl or not (p<.05). C) Microinjection of the JNK inhibitor SP600125 into the ventrolateral PAG with morphine on Trials 1–4 prevented the development of morphine tolerance (F(3,142)  = 13.82, p = .0001). D) Microinjection of SP600125 (100 ng/0.4 µL) with fentanyl on Trials 1–4 prevented the development of fentanyl tolerance (F(3,137)  = 7.866, p = .0001).

The effect of prior administration of Ro 32-0432 or SP600125 on the development of tolerance was assessed 18 hours after Trial 4 by microinjecting cumulative doses of morphine or fentanyl into the ventrolateral PAG. Repeated microinjections of either morphine or fentanyl into the ventrolateral PAG caused tolerance as evident by a rightward shift in the dose-response curve compared to rats treated with saline on Trials 1–4 (Fig. 7). Administration of Ro 32-0432 with morphine on Trials 1–4 prevented the development of morphine tolerance assessed on Trial 5 (Fig. 7A). In contrast, repeated microinjections of Ro 32-0432, whether with fentanyl or not, reduced fentanyl potency as evident by rightward shifts in the dose-response curves (Fig. 7B). Changes in potency (D_50_) as a result of repeated morphine or fentanyl administration with and without Ro 32-0432 are shown in Table 2. In sum, inhibition of GRK/PKC activation attenuated the development of morphine tolerance and inhibited fentanyl antinociception.

**Table 2:** Effect of GRK/PKC and JNK inhibition on the Development of Tolerance.

Protein	Trials 1–4	Morphine D_50_ (N)	Fentanyl D_50_ (N)
GRK/PKC	Vehicle & Saline	7.9 µg (9)	1.7 µg (7)
	Ro 32-0432 & Saline	6.1 µg (8)	*3.1 µg (7)
	Vehicle & Opioid	18.6 µg (9)	3.0 µg (8)
	Ro 32-0432 & Opioid	*8.4 µg (7)	6.3 µg (8)
JNK	Vehicle & Saline	4.2 µg (8)	2.5 µg (6)
	SP600125	4.8 µg (7)	3.6 µg (8)
	Vehicle & Opioid	13.4 µg (7)	7.0 µg (7)
	SP600125 & Opioid	*7.9 µg (8)	*2.0 µg (8)

Notes: N =  sample size.

*Denotes a significant difference from the preceding vehicle group.

Microinjection of SP600125 into the ventrolateral PAG on Trials 1–4 had no effect on morphine or fentanyl antinociception in saline pretreated rats, but attenuated the development of tolerance in morphine (Fig. 7C) and fentanyl (Fig. 7D) pretreated rats. Changes in potency (D_50_) as a result of repeated morphine or fentanyl administration with and without SP600125 are shown in Table 2. These data demonstrate that disrupting JNK signaling prevents the development of both morphine and fentanyl tolerance.

## Discussion

The present data reveal functionally selective mechanisms underlying antinociception and tolerance to morphine and fentanyl microinjections into the ventrolateral PAG. Microinjection of Ro 32-0432 into the ventrolateral PAG to block GRK/PKC phosphorylation of the MOPr had no effect on baseline nociception, enhanced morphine antinociception, and reversed the expression of fentanyl tolerance. Inhibition of JNK signaling by microinjection of SP600125 into the PAG had no effect on baseline nociception or morphine or fentanyl antinociception, but blocked the expression of morphine tolerance and the development of tolerance to both morphine and fentanyl. A summary of these effects is shown in Table 3. The difference in the involvement of GRK/PKC and JNK signaling between the expression of morphine and fentanyl tolerance demonstrate that multiple mechanisms for opioid tolerance exist within the PAG.

**Table 3:** Summary of ligand-biased signaling for morphine and fentanyl antinociception and tolerance following microinjection into the ventrolateral PAG.

	Morphine	Fentanyl
**Antinociception**		
GRK/PKC	Inhibits antinociception	No effect
JNK	No effect	No effect
**Expression of Tolerance**		
GRK/PKC	No effect	Contributes to tolerance
JNK	Contributes to tolerance	No effect
**Development of Tolerance**		
GRK/PKC	Contributes to tolerance	Enhances antinociception
JNK	Contributes to tolerance	Contributes to tolerance

The lack of effect of blocking MOPr phosphorylation or JNK signaling on baseline nociception demonstrates that changes in opioid tolerance are not secondary to changes in nociception. Manipulations that enhance antinociception also can confound interpretation of studies reporting opioid tolerance. For example, our enhancement of morphine antinociception following microinjection of Ro 32-0432 into the PAG could confound the assessment of tolerance. We avoided this problem by assessing the effect of Ro 32-0432 on the development of tolerance 18 hours after the last injection of Ro 32-0432. Moreover, microinjection of Ro 32-0432 occurred immediately prior to the expression of morphine tolerance but had no effect, suggesting that GRK/PKC regulates morphine antinociception specifically. This enhancement was not evident following a single injection of morphine (see Fig. 6). This difference might be caused by GRK/PKC inhibition prolonging antinociception as opposed to increasing the magnitude of antinociception. This prolonged antinociception would be evident with the prolonged cumulative dose response testing, but not following assessment of antinociception 30 min after a single morphine injection. A final point is that the different vehicles for Ro 32-0432 and SP600125 could influence morphine and fentanyl antinociception [22].

The enhanced morphine antinociception following microinjection of Ro 32-0432 into the PAG is consistent with data from *in vitro* studies showing enhanced morphine signaling when MOPr phosphorylation is prevented by blocking GRK and/or PKC activation [14], [23], [24]. Given that MOPr phosphorylation terminates G-protein signaling and G-protein signaling appears to underlie the antinociceptive effects of opioids [13], [25], it is not surprising that blocking GRK/PKC phosphorylation of the MOPr enhanced morphine antinociception. One would expect that blocking GRK/PKC would have a similar effect on fentanyl antinociception, but that was not the case. It is possible that kinases other than those blocked by Ro 32-0432 phosphorylate the MOPr following fentanyl binding or that other signaling pathways, such as those activated by MOPr internalization, contribute to fentanyl antinociception. Whatever the reason for the lack of a change in fentanyl antinociception, the present data clearly show that functionally selective signaling regulates morphine and fentanyl antinociception.

Once tolerance develops to repeated morphine injections, blockade of GRK/PKC activation by microinjection of Ro 32-0432 neither enhanced nor reversed morphine antinociception, whereas the expression of fentanyl tolerance was reversed by Ro 32-0432 administration. Inhibition of JNK activation by microinjecting SP600125 into the PAG had the opposite effect: The expression of morphine, but not fentanyl tolerance was reversed. These findings are consistent with a study by Melief et al. [5] showing that morphine but not fentanyl tolerance was reversed by inhibition of JNK activation, and fentanyl but not morphine tolerance was disrupted in GRK knockout mice. However, other studies report contradictory findings: Inhibition of PKC has been shown to reverse tolerance to continuous morphine administration [26], [27], and intracerebroventricular administration of Ro 32-0432 did not reverse an acute form of fentanyl tolerance [6]. These differences point out that subtle methodological differences such as species (rat vs. mouse), brain region targeted (PAG vs. intracerebroventricular), and method to induce tolerance (repeated microinjections vs. continuous administration) can influence the tolerance mechanism engaged.

Nonetheless, the present data clearly show different mechanisms of opioid tolerance even when the methodology is the same. Both morphine and fentanyl were injected directly into the ventrolateral PAG where they have comparable antinociceptive efficacy [9]. Although the antinociception produced by microinjecting fentanyl into the ventrolateral PAG is more potent and has a shorter duration of action than morphine [7], the magnitude of tolerance with repeated microinjections is similar [9]. The difference in drug duration results in a much longer test session for cumulative doses of morphine compared to fentanyl, and could interfere with the ability of Ro 32-0432 and SP600125 to block morphine effects. However, this long duration did not appear to limit the efficacy of these blockers as indicated by the ability of Ro 32-0432 administration to enhance morphine antinociception and administration of SP600125 to attenuate the expression of morphine tolerance.

In contrast to the expression of tolerance, both GRK/PKC and JNK appear to contribute to the development of morphine tolerance. JNK also contributes to the development of fentanyl tolerance. The only situation in which tolerance was not disrupted was with repeated administration of the GRK/PKC blocker Ro 32-0432 on the development of fentanyl tolerance. Repeated administration of Ro 32-0432 reduced fentanyl antinociception whether rats were tolerant or not (see Fig. 7). Assessment of the development of tolerance requires microinjection of the protein blocker prior to each opioid injection, allowing for greater adaptations than occur with the single injection required to assess the expression of tolerance.

Inhibition of JNK could counteract the development of morphine and fentanyl tolerance by increasing the expression of MOPrs [15], [28]. The opposite effects of repeated inhibition of GRK/PKC on the development of morphine (reverses tolerance) and fentanyl tolerance (blocks antinociception) is harder to explain, but provides a fourth example of ligand-biased signaling in this study. These four examples of ligand biased signaling are:

- Microinjection of Ro 32-0432 attenuates morphine, but not fentanyl antinociception;
- Microinjection of SP600125 inhibits the expression of morphine, but not fentanyl tolerance;
- Microinjection of Ro 32-0432 inhibits the expression of fentanyl, but not morphine tolerance; and
- Repeated microinjections of R0 32-0432 inhibit the development of morphine tolerance and fentanyl antinociception (Table 3).

Such functionally selective signaling is consistent with studies in heterologous cell systems showing differences in morphine and fentanyl signaling [29], [30].

Although our data show that both GRK/PKC and JNK contribute to tolerance depending on the opioid, these molecules are just one part of a complex adaptive process. Blocking proteins anywhere along the signaling pathway that runs from the MOPr to JNK should also prevent the development of opioid tolerance. A decrease in MOPr signaling as a result of enhanced MOPr internalization and degradation combined with a lack of MOPr replacement is a possible mechanism for this tolerance. An increase in GRK signaling has been shown to enhance MOPr internalization [31] and morphine activation of JNK appears to inhibit MOPr expression [15], [28], [32], [33]. However, recent studies showing that PAG microglia contribute to morphine tolerance [34], [35] demonstrate that the mechanisms underlying tolerance, even within the PAG, are complex.

The present data indicate that tolerance mechanisms can be identified through carefully designed studies that target specific neural structures, control for effects on antinociception, and distinguish between the expression and development of tolerance. Moreover, the different effects of GRK/PKC on the development and expression of morphine tolerance demonstrate the importance of distinguishing between these two processes (see Fig. 1).

## Supporting Information

S1 Data Raw data used in this manuscript.
(XLSX)
